# Genetic evolutionary and pathogenicity analyses of a novel porcine reproductive and respiratory syndrome virus 1 strain SC202404 that emerged in Southwestern China

**DOI:** 10.1186/s12917-026-05488-7

**Published:** 2026-04-25

**Authors:** Xue Gao, Yi Qing, Li Luo, Changying Chen, Pingshun Li, Lei Xie, Xiaoxue Xiang, Haohao Lu, Shuo Feng, Yiwen Pei, Runmin Kang, Jifeng Yu, Jie Liu, Zhidong Zhang, Long Zhou

**Affiliations:** 1 Key Laboratory of Veterinary Medicine in Universities of Sichuan Province, College of Animal and Veterinary Sciences, Southwest Minzu University, Chengdu, 610041 China; 2 Chengdu Livestock and Poultry Genetic Resources Protection Center, Chengdu, Sichuan 610081 China; 3 Animal Healthy Disease Service, Chengdu Chia Tai Agro-Industry & Food, Chengdu, Sichuan 610081 China; 4 Sichuan Provincial Key Laboratory of Animal Breeding and Genetics, Sichuan Animal Science Academy, Chengdu, Sichuan 610066 China; 5 Key Laboratory of Ministry of Education and Sichuan Province for Qinghai-Tibetan Plateau Animal Genetic Resource Reservation and Utilization, Chengdu, 610041 China

**Keywords:** Porcine reproductive and respiratory syndrome virus, PRRSV-1, Moderate pathogenicity, Recombination, Transmission

## Abstract

**Background:**

The porcine reproductive and respiratory syndrome virus 1 (PRRSV-1) was first reported in China in 1997, and its prevalence has increased rapidly in recent years. However, information on the genetic evolution and pathogenicity of the newly emerged PRRSV-1 isolates in China remains limited. To further our knowledge about the novel isolate, SC202404 was selected to analyze its potential evolutionary history, regional circulation, and pathogenicity for piglets.

**Methods:**

The PRRSV SC202404 strain was isolated from diseased pigs in Sichuan Province, Southwestern China in 2024. Complete genomic sequence analyses were conducted using the DNASTAR 7.0 software. The phylogenetic relationship of the PRRSV-1 strains was constructed using the maximum likelihood method in MEGA X. The Bayesian molecular divergence time estimation was analyzed using BEAST v10.5.0 software. Recombination events were detected using RDP V4.26 and SIMPLOT software v3.5.1. Five piglets in the challenge group were inoculated intranasally (2 mL) and intramuscularly (1 mL) with SC202404 (1 × 10^5.5^ TCID_50_/mL). Rectal temperatures and clinical symptoms were monitored and scored daily after challenge. The serum was collected for viremia detection by RT‒qPCR and PRRSV-specific antibody levels by ELISA kit. The histopathological examination of tissues were performed using H&E staining.

**Results:**

A genome comparative analysis revealed that SC202404 shares the highest nucleotide similarity (94.5%) with the PRRSV-1 BJEU06-1-like strain TZJ226, which was identified in a pig farm in Henan Province in 2020. A genomic sequence alignment showed that SC202404 has the same unique 25 aa premature termination in GP3, and a consecutive deletion of 6 aa in GP4 as the TZJ226 strain. Evolutionary analyses conducted on ORF5 sequences using Bayesian phylodynamic models estimated the time of emergence of the SC202404 strain as 2014–2015, which is earlier than the emergence of the TZJ226 strain. Moreover, recombination analyses revealed that SC202404 is a minor parent strain of the recombinant virus TZJ226. Therefore, we hypothesize that the SC202404 and TZJ226 strains might share a common evolutionary origin, potentially suggesting a transmission link or regional circulation between Sichuan and Henan provinces. Furthermore, an animal challenge study in piglets showed that SC202404 infection caused a transient fever, and moderate hemorrhagic and interstitial pneumonia, which indicated that the new virus exhibits moderate pathogenicity to piglets.

**Conclusions:**

A novel PRRSV-1 strain, SC202404, with unique molecular markers in GP3 and GP4, was isolated from Sichuan Province in 2024. Animal challenge study in piglets showed that SC202404 is a moderate virulent strain. Our study aids in understanding the genetic evolution of the novel PRRSV-1 strain SC202404 and highlights the importance of preventing and controlling PRRSV-1 strains in China.

**Supplementary Information:**

The online version contains supplementary material available at 10.1186/s12917-026-05488-7.

## Introduction

Porcine reproductive and respiratory syndrome (PRRS), also known as Blue ear Disease, is an economically important disease of swine [1]. Clinical signs of PRRS include reproductive failure in breeding sows, as well as pneumonia and high mortality rates in weaned piglets and growing pigs [2]. The etiological agent, the PRRS virus (PRRSV), is an enveloped virus belonging to the genus *Arterivirus*, family *Arteriviridae*, order *Nidovirales* [3, 4]. Based on antigenicity, PRRSV is divided into two species: PRRSV-1 (species *Betaarterivirus suid 1*) and PRRSV-2 (species *Betaarterivirus suid 2*) [5]. Lelystad virus (LV) and VR-2332 serve as the prototype strains for PRRSV-1 and PRRSV-2, respectively, which share 60% nucleotide identity at the whole-genome level [6, 7].

The genome of PRRSV consists of single-stranded positive-sense RNA, with a length of approximately 15.1–15.5 kb [8, 9], comprising 5ʹ and 3ʹ untranslated regions (UTRs) and 11 open reading frames (ORFs): 1a, 1b, 2a, 2b, 3–5, 5a, 6–7, and a short transframe (TF) ORF in the NSP2 region [10]. The ORFs encode two long polypeptides (PP) of two polyproteins, pp1a and pp1ab, and the following seven structural proteins: the glycoprotein GP2a, small envelope (E) protein, glycoproteins GP3, GP4, GP5, membrane (M) proteins, and nucleocapsid (N) protein [11].

PRRSV-1 was first described as a mysterious pathogen in the swine industry in Europe in 1990. Since then, PRRSV-1 spread throughout European countries and was subsequently reported in Asia, America, and other countries. It has caused devastating economic losses to the global swine industry [12]. To date, four major known lineagesof PRRSV-1 have been identified: lineage 1 (former Global subtype 1), lineage 2 (Russia subtype 1), lineage 3 (subtype 2), and lineage 4 (subtype 3), of which lineage 1 can be further divided into 18 sublineages, L1.1 to L1.18 [13]. Among the four subtypes, only L1 is prevalent globally, whereas other lineages are mainly reported in European countries. In China, PRRSV-1 lineage 1 has been the dominant strain in the field since it was first detected in 1997 [14]. Currently, the majority of the PRRSV-1 lineage 1 strains in China can be divided into four clusters: Amervac-like (L1.2), BJEU06-1-like (L1.13), HKEU16-like (L1) and NMEU09-like (L1.10) [13]. The BJEU06-1-like strain was first reported in China in 2006 and has become the predominant circulating strain in recent years [15].

In 2020, a TZJ-226 (BJEU06-1-like) strain, was isolated from a pig farm in Henan Province. Notably, the virus contains a unique 25 aa premature termination in ORF3, and a continuous deletion of 6 aa in ORF4 [15]. The PRRSV-1 strain SC202404 was isolated from a pig-finishing farm in Sichuan Province in 2024. Interestingly, the genome of SC202404 shared the highest nucleotide sequence identity with TZJ226 and exhibited the same molecular markers in ORF3 and ORF4. The aim of our study was to analyze the evolutionary process and pathogenicity of the PRRSV-1 strain isolated in Sichuan Province.

## Materials and methods

### Sample collection and virus isolation

In 2024, approximately 25% of piglets (30–48 days old) in a commercial pig farm (750 head) in Sichuan Province presented with high fever, accelerated respiration, and acute decreases in appetite and activity. Since no cases of PRRS had been found on this farm, the pigs were never vaccinated with PRRSV-related vaccines. Blood samples from 10 diseased piglets suffering from severe dyspnea were gathered and brought immediately to the laboratory.

These samples were centrifuged at 3000 rpm for 10 min at 4 °C, and the sera were tested for PRRSV-1, PRRSV-2, classical swine fever virus (CSFV), porcine circovirus (PCV), and pseudorabies virus (PRV) using PCR or RT-PCR [16]. The primers designed to detect different viruses are shown in Supplementary Table S1. Viral RNA was extracted from serum using TRIzol reagent (Invitrogen, Carlsbad, CA, USA). Then the RNA was used to synthesize cDNA using a cDNA Synthesis Kit (TaKaRa, Dalian, China). PRRSV-positive samples were filtered through a 0.22-μm filter and transferred to pulmonary alveolar macrophages (PAMs). The PAMs were confirmed to be negative for major swine pathogens, including PRRSV, pseudorabies virus (PRV), porcine circovirus type 2 (PCV2), classical swine fever virus (CSFV), and *Mycoplasma hyopneumoniae*. Cells were grown in RPMI-1640 medium supplemented with 10% fetal bovine serum (FBS; VivaCell, Shanghai, China) at 37 °C in a 5% CO_2_ incubator. Cell cultures were monitored daily for cytopathic effects (CPEs) [17]. PRRSV antigen-positive cells were identified by indirect immunofluorescence (IFA) assays using a PRRSV N protein-specific monoclonal antibody (GeneTex Inc., Irvine, CA, USA). The isolate was purified by plaque assay as previously described [18], and the virus titers were calculated in accordance with the Reed–Muench method.

### Genome sequencing

Overlapping PCR fragments encompassing the full-length sequence of the PRRSV genome were amplified using 10 pairs of primers listed in Supplementary Table S1. PCR amplification was performed using high-fidelity polymerase Platinum SuperFi II DNA (Invitrogen, USA). The amplified PCR products were purified by a gel extraction kit (OMEGA, USA) and sequenced by the Tsingke commercial service (Chendu, China). The full-length genome sequence was assembled in Lasergene SeqMan 7.1.0 software (DNASTAR, Madison, WI, USA) and then deposited in the GenBank database (https://www.ncbi.nlm.nih.gov/genbank/).

### Genome alignment and phylogenetic analysis

A sequence alignment of the PRRSV ORF5 gene nucleotide sequences between the SC202404 with 969 reference strains from various countries was performed using Clustal W in MEGA X software [13]. The phylogenetic relationship of the nucleotide sequences of PRRSV strains was constructed using the maximum likelihood method with 1,000 bootstrap replicates in MEGA X. The generated phylogenetic tree was annotated using the online software TVBot (https://www.chiplot.online/normalTree). To further characterize the amino acid mutation in Nsp2, GP3, and GP4, amino acid sequences of the three regions were aligned with EuroPRRSV, LV, Amervac PRRS, NMEU09-1, HKEU16, Lena, NVDC-NM2, HK10, BJEU06-1, ZD-1, TZJ226, and TZJ637. Multiple sequence comparisons at the nucleotide and amino acid levels were aligned using ClustalW of DNASTAR’s Lasergene 7.1 software (DNASTAR Inc.) [19].

### Recombination analysis

To test the role of recombination in the generation of the isolated SC202404, the multiple alignments of the genomes were submitted to the Recombination Detection Program 4 (RDP4, version 4.26) to screen for potential recombination events. Recombination events were detected using RDP4 with nine different algorithms (RDP, GENECONV, BootScan, MaxChi, Chimaera, SiScan, 3Seq, LARD, and PhylPro), and at least five methods with a significant *P* value (*P* < 0.05) were taken as significant evidence for recombination. Recombination events were further confirmed and presented by the SimPlot software v.3.5.1 [20].

### Bayesian molecular divergence time estimation

MEGA X software was used to perform the CLUSTAL W alignment on the PRRSV-1 sequences. An XML input file was then generated for BEAST v10.5.0 using BEAUti v10.5.0. BEAUti (within the BEAST package) was used to set the criteria for the analysis, applying a general time reversible (GTR) nucleotide-substitution model with Gamma + Invariant sites, and specifying the proportion of invariant sites [21]. MCMC chains were run for 100,000,000 generations, with parameter values and trees sampled every 1,000 generations to ensure convergence (effective sample sizes [ESS] > 200). The BEAST log files were inspected for convergence using Tracer v.1.7.2. Trees were annotated using TreeAnnotator v10.5.0 and visualised in FigTree v1.4.4 [22, 23].

### Animal experiments

Ten 4-week-old PRRSV-naive piglets were purchased from a farm in Sichuan province. The pigs were confirmed negative for PRRSV, PRV, PCV2, and CSFV by PCR/RT-PCR on nasal swabs. The pigs were also determined to be negative for PRRSV antibodies using a commercial ELISA kit (IDEXX HerdChek ELISA, Westbrook, USA) on serum samples. All pigs were weighed and randomly divided into 2 groups: a challenge group (*n* = 5) and a negative control group (*n* = 5). Each group was housed separately in two different rooms. Piglets in the PRRSV challenge group were inoculated intranasally (2 mL) and intramuscularly (1 mL) with SC202404 (1 × 10^5.5^ TCID_50_/mL, 3 mL/pig). The piglets in the control group were administered a mock inoculation of 3 ml of uninfected RPMI 1640. Rectal temperatures and clinical symptoms were monitored and scored daily. The serum was collected at 0, 3, 5, 7, 10, and 14 days post-inoculation (dpi) from pigs for viremia detection by real-time quantitative PCR (RT‒qPCR). All piglets were weighed weekly and all piglets were humanely euthanized at 14 days post-infection (dpi) with an intravenous overdose of sodium pentobarbital (Sigma-Alrdich, 100 mg/kg). Death was confirmed by the cessation of a palpable heartbeat and respiratory arrest for a minimum of 5 min. This method aligns with the American Veterinary Medical Association (AVMA) Guidelines, ensuring a rapid and painless death while minimizing animal distress; the carcasses were subjected to harmless treatment. In addition, tissue samples, including heart, liver, spleen, lung, kidney, tonsil, and lymph node, were collected. Portions of tissue samples were immersed in 4% paraformaldehyde for histopathological and immunohistochemical (IHC) examinations as previously described [18]. The NSP2 gene from the tissue samples was amplified and sequenced to confirm that it was the original virus. The study was approved by the Southwest Minzu University Institutional Animal Care and Use Committee (registration protocol SMU-202401058).

### Viral detection by RT‑qPCR

Viremia and viral loads in tissues were quantified by RT-qPCR assay using a primer targeting the NSP2 of PRRSV-1 (Supplementary Table S2). A standard curve generated using a serial ten-fold diluted (10^9^ to 10^4^ copies/μL) plasmid containing the NSP2 was used for absolute quantification. Approximately 0.5 g of tissue homogenate was prepared from the tissue samples using 1 mL of RPMI-1640 medium. The supernatant was collected after centrifugation at 10,000 × g for 8 min at 4℃ to remove residual tissue debris [24]. Each serum sample was centrifuged at 3000 rpm for 10 min at 4 °C to collect serum. Total RNA was extracted by TRIzol Reagent (Invitrogen, USA) and reverse transcribed to cDNA using Reverse Transcriptase (TOYOBO, Shanghai, China). Viral RNA loads in serum and tissues were calculated according to the standard curve [25].

### Detection of PRRSV antibodies

Per the manufacturer's instructions, PRRSV-specific antibodies were measured using a commercial ELISA kit (IDEXX, Inc., Westbrook, ME, USA). Based on the manufacturer’s guidelines, sample-to-positive control (S/P) ratios greater than 0.4 were considered positive for PRRSV antibody.

### Statistical analysis

All data were expressed as means ± standard deviations (SD). Statistical significance between the two groups was ascertained by conducting t-tests with GraphPad Prism software version 10 (San Diego, CA, USA), where a p-value less than 0.05 was deemed to indicate statistical significance.

## Results

### Isolation and identification of PRRSV

**Fig. 1:**
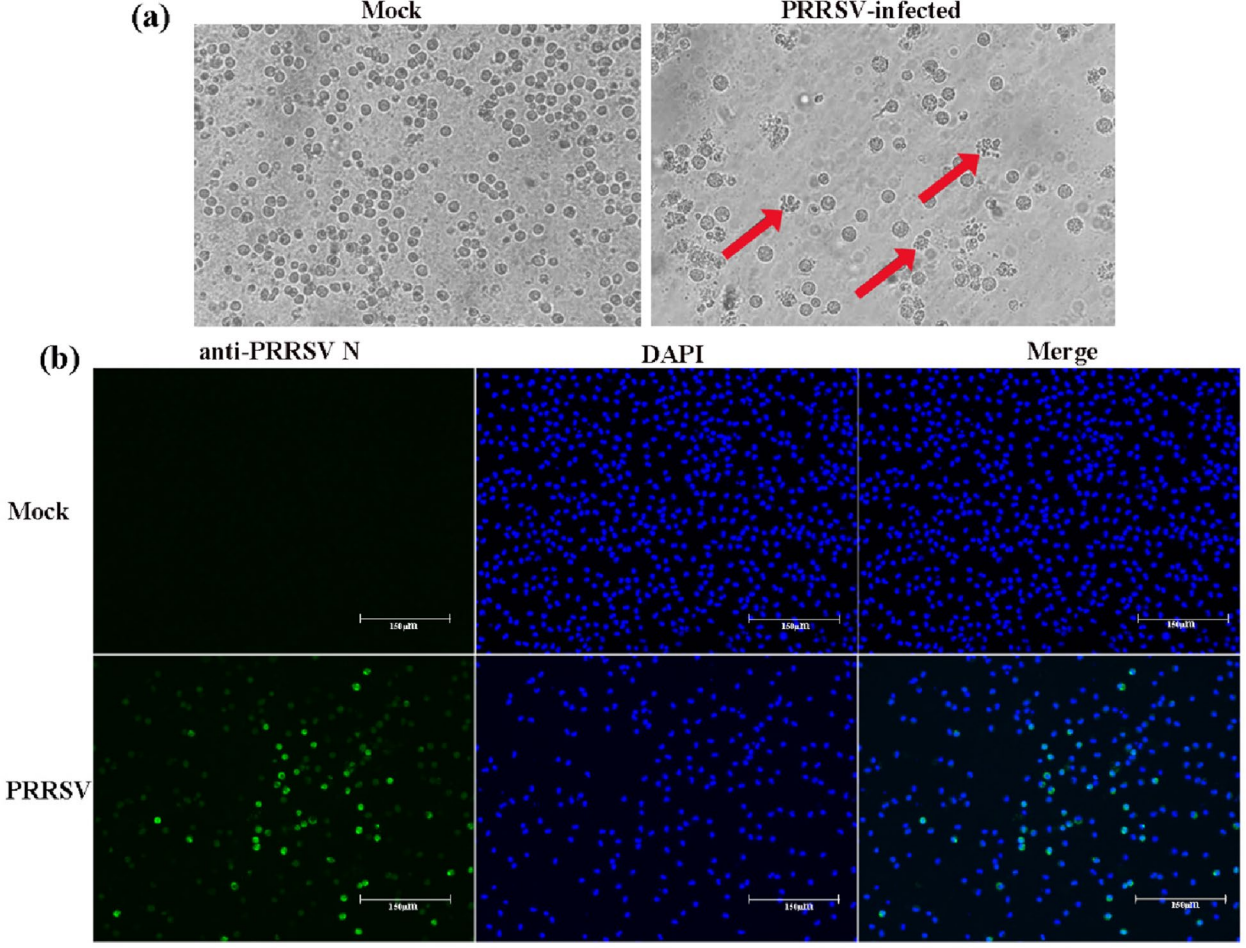
Viral isolation of PRRSV SC202404. **a** PAMs were inoculated with PRRSV GX2024 as shown at 48 h postinfection; (**b**) PAMs were infected with six passage viral cultures for 24 h and examined by IFA with the anti-N PRRSV monoclonal antibody. Cell nuclei are stained with DAPI. Scale bar = 150 μm

Before virus isolation, the samples were subjected to PCR/RT-PCR to detect potential viruses. Of the 10 serum samples, 9 (90%) were positive for PRRSV, but negative for CSFV, PCV, and PRV. The PRRSV-positive samples were utilized for isolating the virus. As illustrated in Fig. 1a, the infected PAMs exhibited significant CPEs, including cell collapsing and shedding, at 48 h after viral inoculation. No obvious CPEs were observed in the uninfected cells (Fig. 1a). The isolated virus was designated strain SC202404 and underwent six blind passages in PAMs. The viral supernatants from the 6th passage were tested using RT-PCR to confirm positivity for PRRSV. At 24 h post-infection (hpi), IFA confirmed the presence of the N protein of PRRSV-1 in the infected PAMs, where it was not observable in mock-infected cells (Fig. 1b). The results confirmed the successful isolation of the SC202404 strain. Additionally, in PAMs, the TCID_50_ value of the SC202404 strain was determined as 1 × 10^5.5^/mL.

### Phylogenetic analysis

**Fig. 2:**
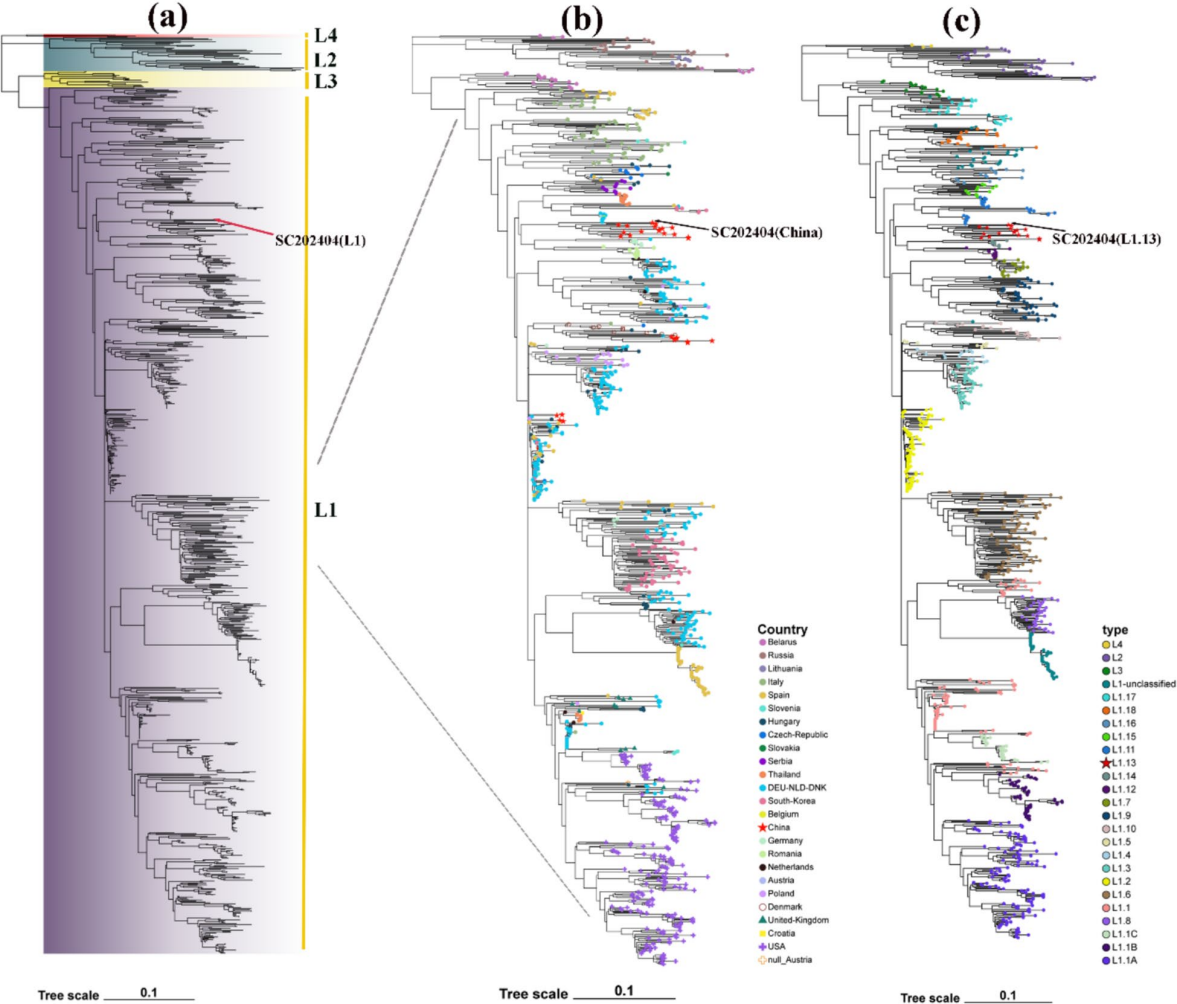
Phylogenetic tree demonstrating PRRSV-1 ORF5-based genetic classification. **a** Maximum-likelihood trees of L1-L4 PRRSVs based on ORF5 sequences. **b** and **c** Geographical distribution and lineage (sublineage) of PRRSV-1 are labelled with solid circles filled with different colors. SC202404 and L1.13 are marked with a red pentagram

Based on analysis of 969 global ORF5 sequences of strains from 25 countries reported in during 1990–2024, PRRSV-1 strains were classified into four lineages (L1–L4) and L1 strains were further divided into 18 sublineages (L1.1–L.18). The overwhelming majority of the PRRSV-1 strains belonged to L1 (*n* = 913; 94.22%) and were widely prevalent in 22 countries. Only a few sequences belonged to L2 (*n* = 36; 3.72%, Russia, Lithuania, and Belarus), L3 (*n* = 17; 1.75%, Belarus), or L4 (*n* = 3; 0.3%, Belarus). In China, the PRRSV-1 isolates were mainly divided into L1.13 (*n* = 17; 1.75%), L1.10 (*n* = 7; 0.72%), and L1.2 (*n* = 5; 0.52%). To understand the genetic relationships between known strains and the newly identified PRRSV-1 isolate SC202404, a phylogenetic analysis was performed. It showed that SC202404 clustered with mostly PRRSV-1 strains from China. It was classified as L1 (Fig. 2a and b) and assigned to L1.13 (Fig. 2c), based on the phylogenetic analysis.

### Genomic characteristics of SC202404

**Fig. 3:**
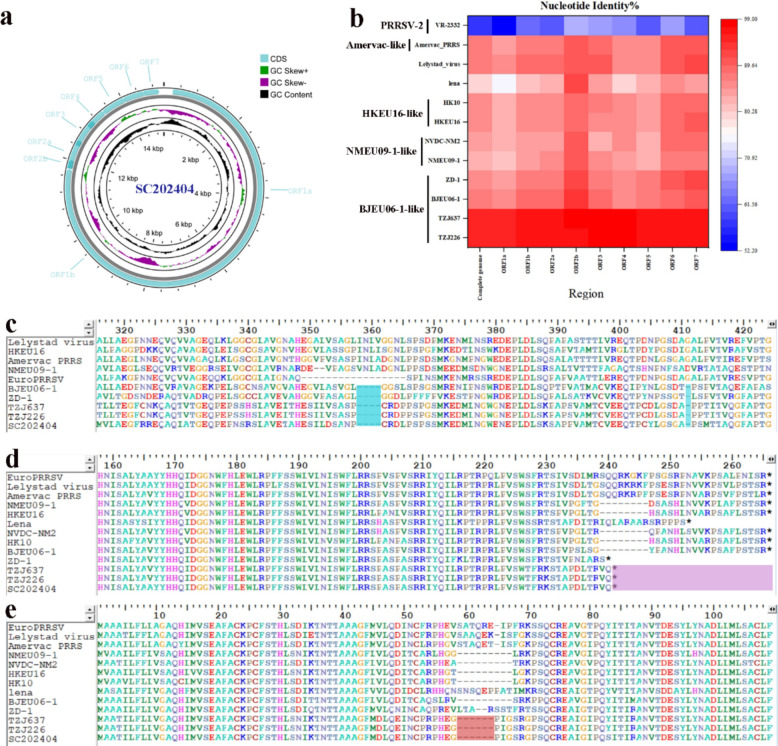
Genomic analysis of SC202404. **a** Whole genome of SC202404 isolate; (**b**) Nucleotide identity of SC202404 compared with twelve PRRSV reference strains; (**c**) Genetic characterization of the SC202404 strain. SC202404 clusters within the BJEU06-1-like branch and shares a 4-aa (358–361) and a 1-aa (411) deletion in NSP2 (blue regions). **d** The strain also exhibits a 25-aa early termination in GP3 (purple regions) (**e**) and a consecutive 6-aa deletion (59–64) in GP4 (red regions)

The full-length genome of SC202404 comprises 15,068 nt, excluding the poly (A) tail, with a 52.29% GC content (Fig. 3a). The sequences were deposited in the GenBank database under the accession number PV533618. A comparative sequence analysis showed that the genome of SC202404 shared 88.0%, 83.4%, 85.2%, and 86.7% sequence identities with BJEU06-1, NMEU09-1, HKEU16, and Amervac PRRSV, respectively, but only a 58.2% nucleotide identity with the North American prototype VR-2332 (PRRSV-2). The overall results indicated that the SC202404 strain belongs to PRRSV-1. Furthermore, the genome of the SC202404 isolate was compared with 11 PRRSV-1 representative strains (TZJ226, TZJ637, BJEU06-1, ZD-1, NMEU09-1, NVDC-NM2, HKEU16, HK10, Amervac PRRSV, Lelystad, and lena), and 1 PRRSV-2 representative strain (VR-2332). The result showed that the genome of SC202404 shared the highest nucleotide similarity (94.5%) with TZJ226 and TZJ637 strains (TZJ226 and TZJ637 are the first and fifth isolates, respectively, from a pig farm in Henan Province, taken at 10 time periods). Additionally, the ORF1a and ORF5 of SC202404 shared 95.6% and 96.3% nucleotide (nt) similarity levels with the TZJ226 strain, These were higher than the similarities shared with the other strains. ORF1b–ORF2b of SC202404 shared 96.3–97.7% nucleotide similarity with the TZJ637 strain. The SC202404 isolate shared 96.2–98.9% nucleotide similarity with both the TZJ226 and TZJ637 strains in the ORF3–ORF4 and ORF6–ORF7 regions. (Fig. 3b).

Each fragment of the SC202404 genome was compared with corresponding fragments of other PRRSV-1 representative strains, and characteristic changes were identified in NSP2, GP3, and GP4 (Fig. 3). For NSP2, SC202404 is similar to four BJEU06-1-like strains (BJEU06-1, ZD-1, TZJ226, and TZJ637), having a 4-aa deletion between aa 358 and 361 and a 1-aa deletion at position 411 of NSP2 (Fig. 3c). Interestingly, the structural protein encoded by ORF3 in SC202404 showed a unique 25-aa premature termination, which was also identified in TZJ226 and TZJ637 strains in 2020 (Fig. 3d). Moreover, a consecutive deletion of 6-aa from positions 59 to 64 of GP4 was found in SC202404, which is consistent with the deletions found in the TZJ226 and TZJ637 strains (Fig. 3e).

### Recombination analysis

**Fig. 4:**
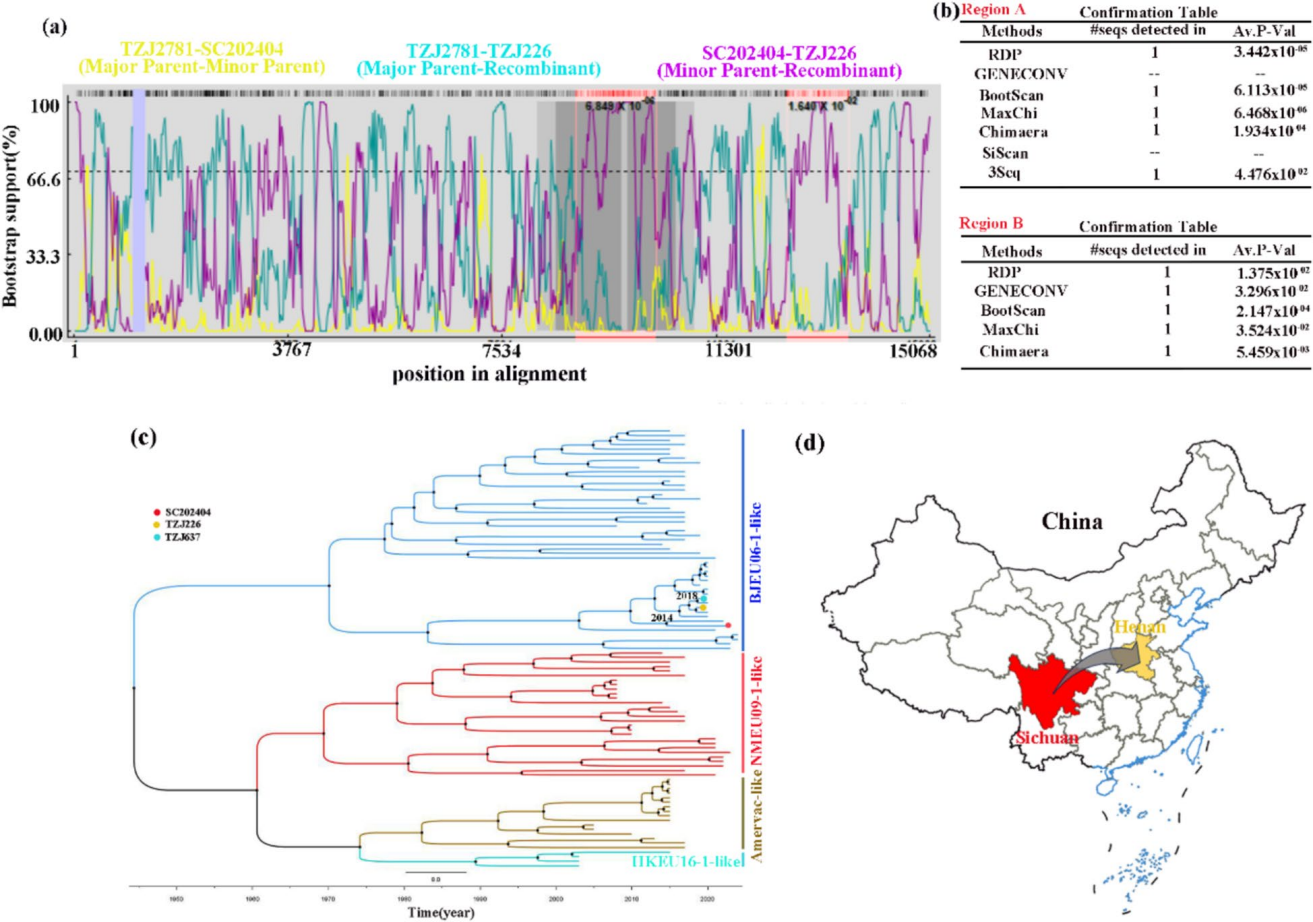
Genome recombination analysis of the SC202404 isolate, and Bayesian inference of SC202404 transmission dynamics in China. **a** Genome recombination analysis of the TZJ226 isolate. The y-axis indicates the percentage similarity between the query sequence (TZJ226) and two representative sequences. Genome-scale similarity comparisons of TZJ226 (recombinat) with SC202404 (red) and TZJ2781 (blue). The supposed recombination regions are shown with two yellow shadows. The recombination breakpoints are marked at the bottom with nucleotide sites, and the viral genome structure is referenced to VR-2332. **b** Recombination detection in Regions A and B. A P-value of less than 0.05 was considered statistically significant. **c** PRRSV-1 differentiation time of Bayesian software estimation. SC202404 strain sequences are marked with a red circle, TZJ226 strain sequences are marked with a yellow circle, TZJ637 strain sequences are marked with a blue circle. **d** Geographical spread of SC202404 and TZJ226 strains in China

We examined the sequence alignment among SC202404, TZJ226 and TZJ2781 strains using RDP4 and SimPlot to identify potential recombination events. The recombination analysis revealed that TZJ226 is a natural recombinant virus of TZJ2781 (major parental strain) and SC202404 (minor parental strain) (Fig. 4a). Similarity plotting identified two recombination regions (Regions A and B) and four recombination breakpoints (nt 8,848, nt 10,250, nt 12,542, and nt 13,634) in the TZJ226 genome. For Region A, five methods (RDP4, BootScan, MaxChi, Chimaera, and 3Seq) were used to detect significant evidence of recombination (*P* < 0.05). For Region B, five methods (RDP4, GENECONV, BootScan, MaxChi, and Chimaera) were used todetect significant evidence of recombination (*P* < 0.05) (Fig. 4b). Therefore, the TZJ226 strain likely originated from a recombination event between the SC202404 and TZJ2781 strains.

### Divergence-time estimation for PRRSV-1 TZJ226 and SC202404 strains

For the divergence-time estimation analysis, 97 sequences of the PRRSV-1 strains were obtained from the NCBI database, and the sequences of the target strains were also included. After the analysis, a multiple sequence alignment using MEGA was performed. Subsequently, BEAST was used to reconstruct their phylogenetic tree and estimate divergence times. The tree topology obtained using a Bayesian analysis was similar to the one obtained using the Maximum-likelihood method. A phylogenetic chronogram of lineage divergence in the PRRSV-1 strains based on a relaxed molecular clock suggested the emergence of the SC202404 strain in China in 2014–2015, about four years earlier than the TZJ226 strain (2018–2019) (Fig. 4c). Therefore, we hypothesize that the SC202404 and TZJ226 strains might share a common evolutionary origin, potentially suggesting a transmission link or regional circulation between Sichuan and Henan provinces (Fig. 4d).

### Clinical signs of SC202404 infected in piglets

**Fig. 5:**
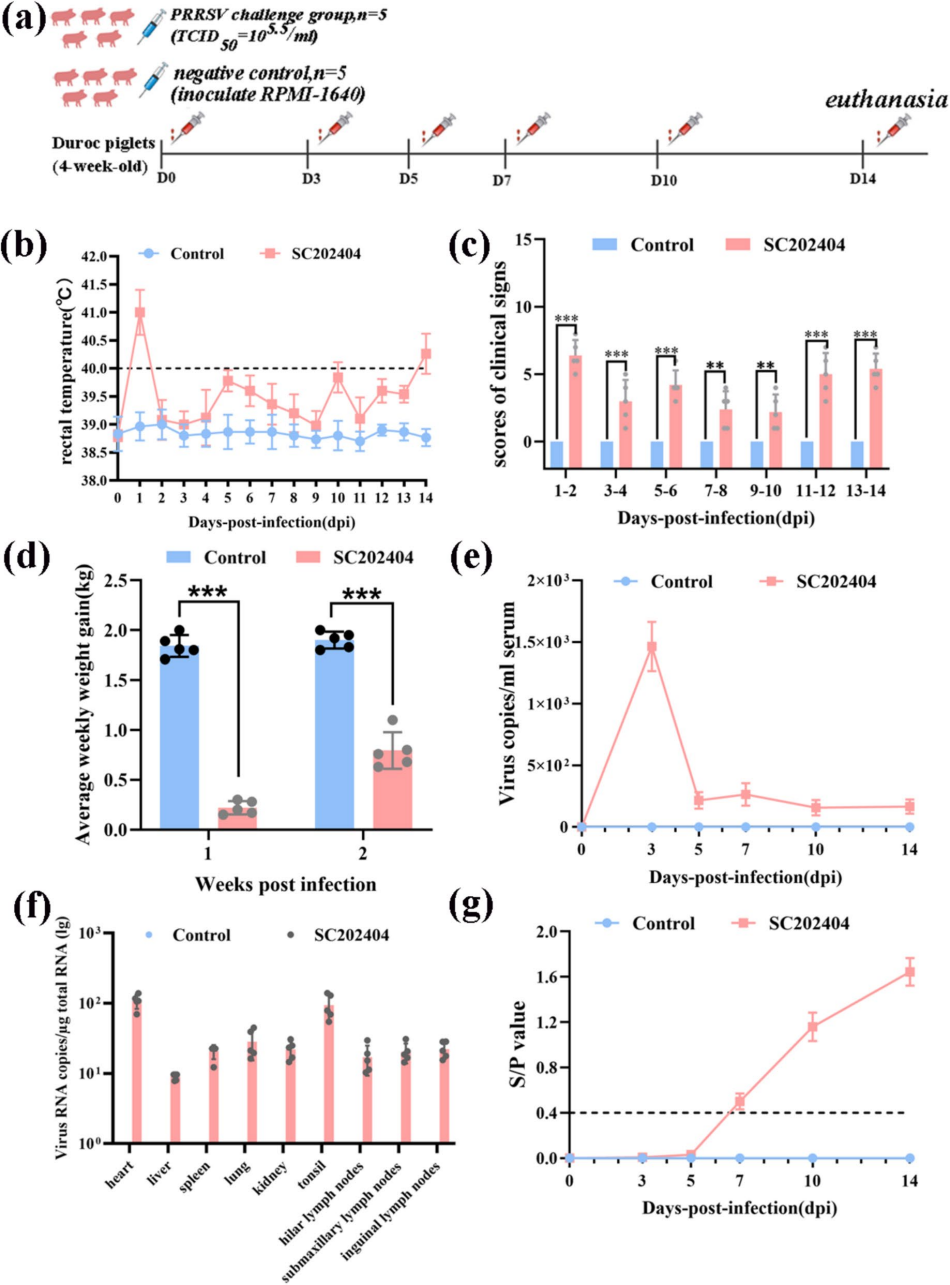
Pathogenicity analysis of the PRRSV isolate SC202404 in piglets. **a** The animal experimental design of this study. **b** Rectal temperatures of pigs inoculated with the SC202404 isolate and RPMI-1640 medium. The clinical fever cut-off value was set at 40.0 °C. **c** The scores of clinical signs of the challenge study. **d** Average weekly weight gain of the inoculated pigs during the challenge experiment. **e** The virus level in serum. **f** PRRSV RNA viral load in pig tissues inoculated after virus attack. **g** Serum samples were collected at 0, 3, 5, 7, 10, and 14 dpi to measure antibodies against the PRRSV N protein. The measured values in this study were expressed as the mean ± standard deviations (SD). Asterisk (*) indicates significant differences between the SC202404 and RPMI-1640 medium inoculated groups (*p < 0.05; **p < 0.01; ***p < 0.001)

The experimental design for the animal infection study is shown in Fig. 5a; briefly, each piglet in the PRRSV group was challenged with the PRRSV-SC202404 strain at a dose of 1.0 × 10^5.5^ TCID₅₀/mL, while those in the control group were inoculated with an equal volume of RPMI-1640. During the challenge period, piglets in the SC202404-infected group developed a high fever (above 41.0 °C) at 1 dpi. Subsequently, their average body temperature rapidly decreased to below 40 °C at 2 dpi. From 2 to 13 dpi, the temperature remained below 40.0 °C. However, the temperature increased again to above 40.0 °C at 14 dpi (Fig. 5b). At 1 dpi, the piglets challenged with PRRSV exhibited disease signs, such as lethargy, fever, and anorexia. By 4 dpi, the SC202404-infected group showed clinical symptoms, including sneezing. By 6 dpi, the piglets challenged with PRRSV showed worsening disease signs of diarrhea, muscle tremors, sneeze, anorexia, and mild ataxia. Overall, the average clinical score of pigs in the PRRSV challenge group was significantly higher than that in the RPMI-1640 group (Fig. 5c). No animals died during the entire experimental period. All the surviving piglets were humanely euthanized at 14 dpi. At both week 1 and week 2 post-infection, the mean average weekly weight gain in the SC202404-infected group were significantly lower than those of the control group (Fig. 5d). The mock-inoculated pigs maintained a body temperature within the normal range throughout the study and exhibited no clinical symptoms or obvious macroscopic lesions.

### Viral loads in sera and tissues

The viral loads in sera and various tissues, including the heart, liver, spleen, lung, kidney, tonsils, hilar lymph nodes, submaxillary lymph nodes, inguinal lymph nodes, and mesenteric lymph nodes, were determined. As shown in Fig. 5, the viremia of SC202404-infected piglets increased significantly by 3 dpi, reached a peak, and then decreased rapidly at 5 dpi (Fig. 5e). Viral loads were also detected in various tissues examined, with the highest levels observed in the heart, followed by the tonsil and the lung (Fig. 5f). The mock-inoculated pigs remained PRRSV PCR-negative during the entire study.

### Antibody detection post-infection

The PRRSV ELISA antibody results are shown in Fig. 5g. For the SC202404-infected group, sera samples tested negative at 0, 3, and 5 dpi. Seroconversion occurred between 7 and 14 dpi, by which time 100% (5/5) of the piglets had become seropositive (S/*P* > 0.4). In contrast, all the pigs in the mock-inoculated group remained seronegative throughout the experiment (*S/P* < 0.4) (Fig. 5g).

### Macroscopic and histopathological lesions

#### Gross pathological findings

**Fig. 6:**
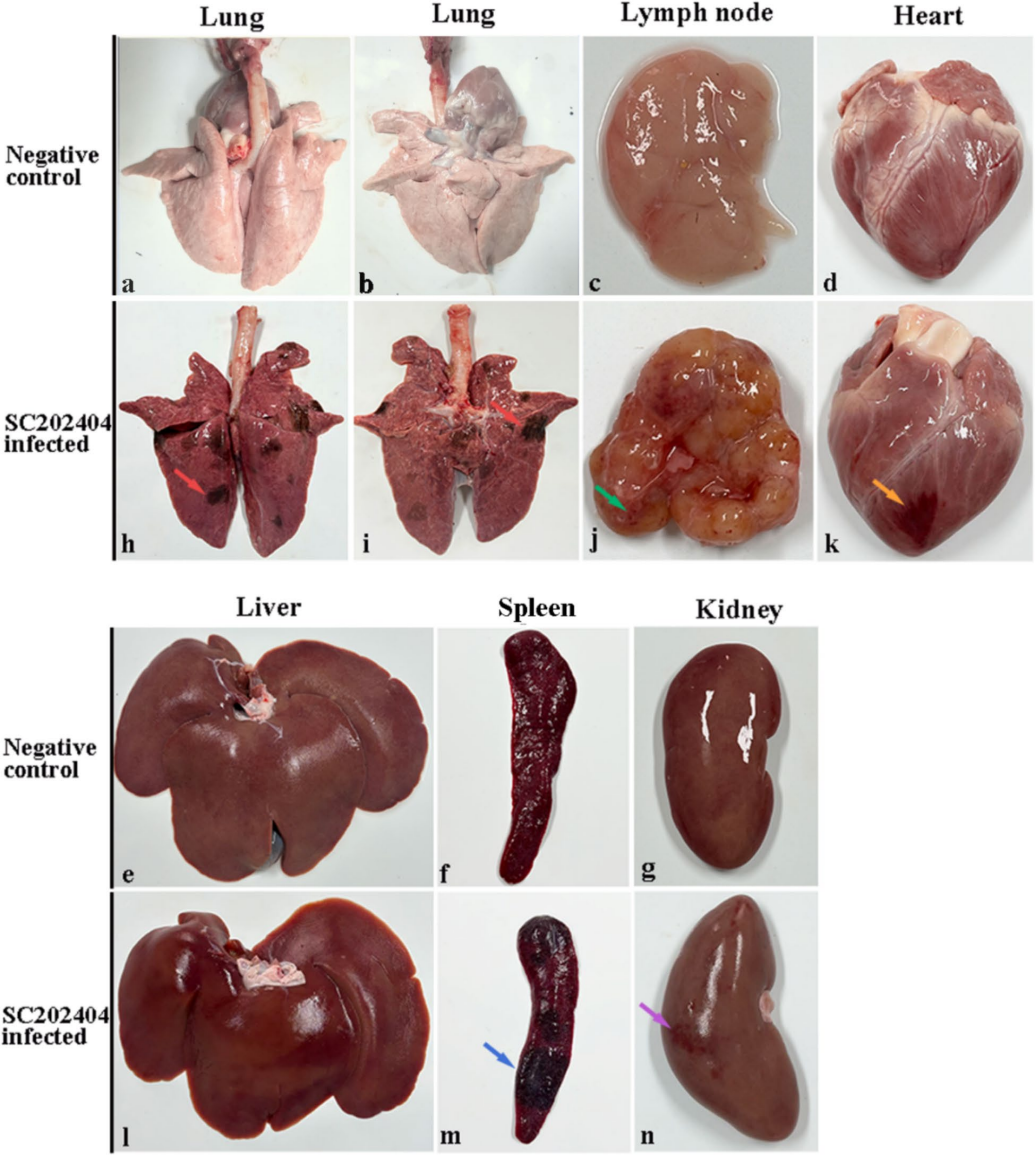
Tissue lesions observation of the inoculated piglets. **h**, **i **The major macroscopic lesions observed in the SC202404 challenge group included hemorrhagic and focally necrotic lung tissue (red arrow). **j** Mild hemorrhage in the submaxillary lymph nodes (green arrow). **k** cardiac hemorrhage (orange arrow). **l** uneven discoloration of the liver. **m** Splenic infarction was observed (blue arrow). **n** along with renal ecchymoses (purple arrow). **a-g** No macroscopic lesions were observed in the negative control group

At the end of the experiment, all the pigs were humanely euthanized and necropsied. The major macroscopic lesions observed in the SC202404 challenge group were characterized by multiple lung hemorrhagic spots and interstitial pneumonia (Fig. 6h, i). Additional findings consisted of mild hemorrhages in the submaxillary lymph nodes (Fig. 6j), apical hemorrhages (Fig. 6k), and uneven discolorations of the liver (Fig. 6l). Furthermore, obvious splenic hemorrhages were observed (Fig. 6m), along with renal ecchymoses (Fig. 6n). No macroscopic lesions were observed in the negative control group (Fig. 6a, g).

#### Histopathological analysis (H&E)

**Fig. 7:**
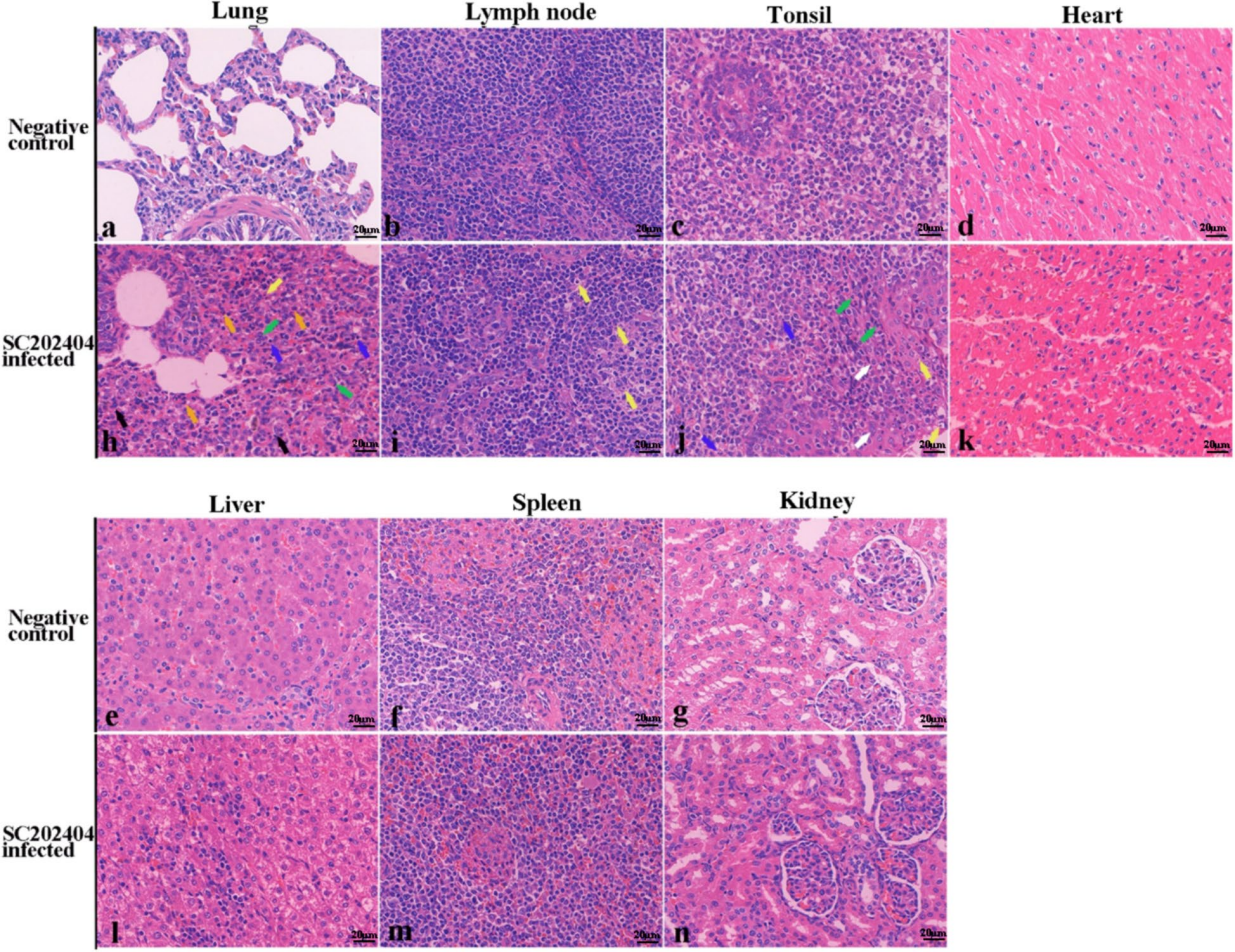
The results of H&E staining in the tissues in each group. **h** The SC202404 challenge group exhibited extensive necrosis of alveolar epithelial cells (yellow arrow) with adjacent neutrophilic (orange arrow) and lymphocytic infiltration (blue arrow), prominent inflammatory cell accumulation, and marked fibrous connective tissue hyperplasia with fibroblast proliferation (black arrow). **i** In the lymph nodes, a small number of lymphocytes in the SC202404 challenge group showed degeneration and necrosis(yellow arrow). **j** Tonsillar abscess formation featuring crypt epithelial cell degeneration and necrosis (yellow arrow), epithelial hyperplasia (white arrow), lymphocytic damage (blue arrow), and neutrophilic infiltration(green arrow). **k**-**n** A large number of infiltrated inflammatory cells were observed in the other tissue. **a-g** Normal histology was determined in the tissue of the negative control group. Original magnification, 400 ×

The histopathological examination of lung tissue using H&E staining revealed that the SC202404 challenge group exhibited prominent inflammatory cell infiltration and fibrous connective tissue hyperplasia. The extensive necrosis of alveolar epithelial cells was observed, accompanied by proliferative changes and accumulations of numerous neutrophils and lymphocytes adjacent to necrotic areas. Additionally, marked fibrous tissue hyperplasia was evident, with a notable increase in fibroblast numbers (Fig. 7h). In lymph nodes, a small number of lymphocytes in the SC202404 challenge group showed degeneration and necrosis (Fig. 7i). The tonsils in this group exhibited abscess formation, characterized by the degeneration and necrosis of crypt epithelial cells, epithelial hyperplasia, lymphocytic degeneration and necrosis, and enhanced neutrophil infiltration (Fig. 7j). Large numbers of infiltrated inflammatory cells were also observed in heart, liver, spleen, and kidney (Fig. 7k–n). Normal histology was determined in the tissues of the mock-inoculated group (Figs. 6g-7a).

#### Immunohistochemical analysis

**Fig. 8:**
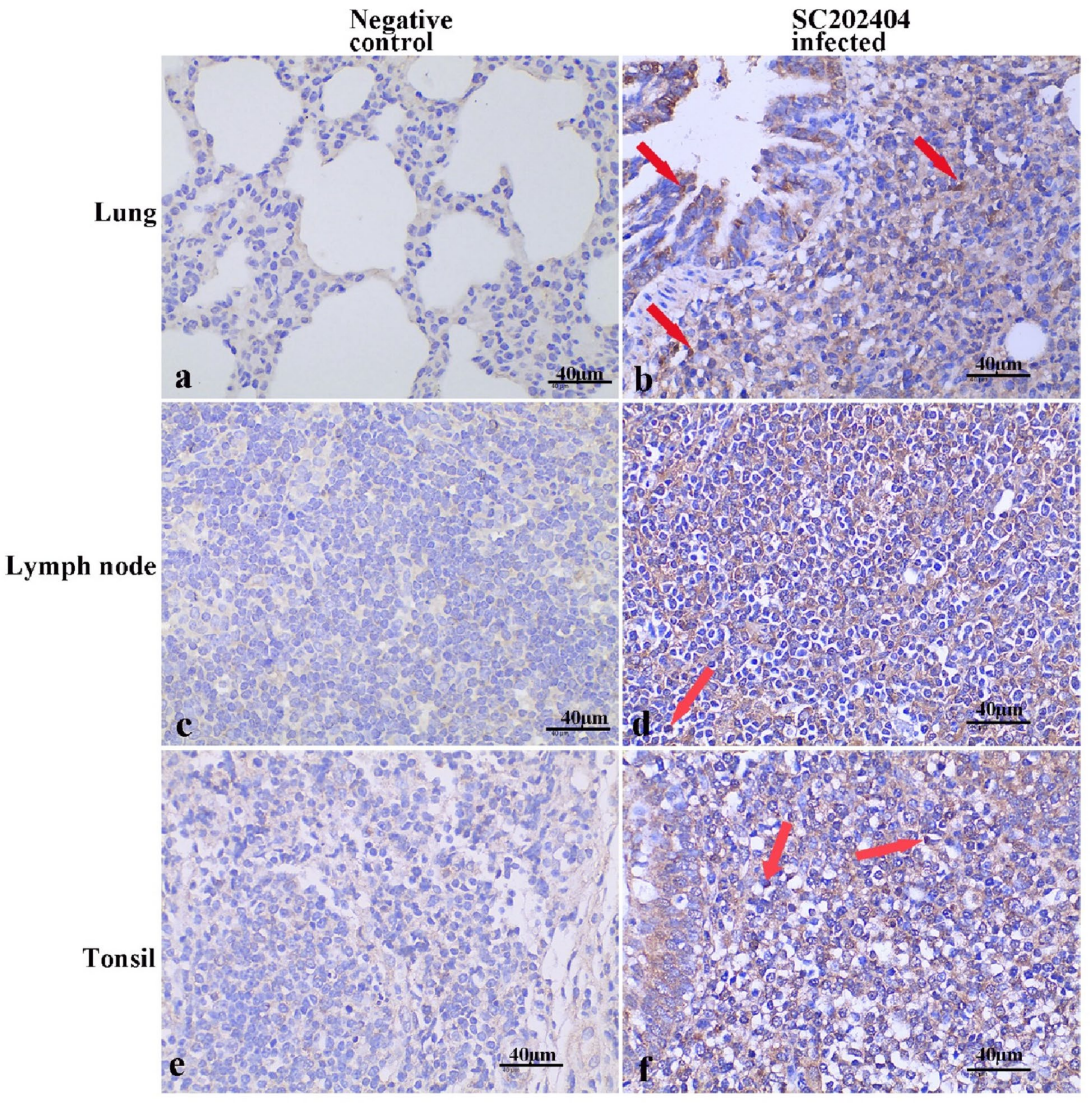
IHC detection of PRRSV antigen. **b** PRRSV-specific staining (brownish-yellow, cytoplasmic/nuclear) is present in the lung, (**d**) lymph node, (**f**) and tonsil of challenged animals. **a**, **c**, **e** No signal is detected in the negative controls. Original magnification, 400 ×

The lung, lymph node, and tonsil tissues were also examined for PRRSV-specific immunohistochemical staining. The PRRSV antigen was mainly detected in the bronchial epithelial cells and the macrophages in lungs (Fig. 8b), lymph nodes (Fig. 8e), and tonsils (Fig. 8h) of pigs in the SC202404-infected group. Positive staining was primarily characterized by brownish-yellow coloration in the cytoplasm and cell nuclei. No positive signals were observed in the mock-inoculated group (Fig. 8a, d, g).

## Discussions

PRRS is a devastating disease that has had a disastrous impact on the global swine industry [26]. In China, PRRSV-2 has been predominant for more than 20 years, since it was first identified in 1995; consequently, whereas PRRSV-1 has always received less attention [27]. However, this is changing due to PRRSV-1’s increased detection rate in the field in recent years. To date, PRRSV-1 strains have spread to more than 23 provinces/autonomous regions in China [28]. Most PRRSV-1 strains in China are classified as L1.13 (BJEU06-1-like strains), L1.2 (Amervac-like strains), L1 (HKEU16-like strains), and L1.10 (NMEU09-like strains) [15]. In this study, a new PRRSV strain, SC202404, was isolated from a pig farm in Sichuan Province, Southwestern China. Genome comparative and phylogenetic analyse revealed that SC202404 shares the highest nucleotide similarity (94.5%) with the BJEU06-1-like strain TZJ226, which was identified in a pig farm in Henan Province in 2020. To our knowledge, this is the first report of a BJEU06-1-like PRRSV-1 strain in Sichuan Province.

Mutation, indel, and recombination events are important mechanisms in PRRSV evolution [29–32]. The *Nsp*2 gene is highly variable and frequently used as a molecular marker to monitor the evolution of PRRSV strains. BJEU06-1-like strains have a unique discontinuous deletion of 5 aa (4 + 1) in the nsp2-coding region relative to the sequence of the Lelystad strain. Additionally, mutation and deletion events frequently occurred in the hypervariable regions of GP3 and GP4 in the PRRSV-1 isolates [33]. An amino acid alignment of the highly variable regions of Nsp2, GP3, and GP4 in PRRSV-1 strains revealed that SC202404 had a discontinuous deletion of 5-aa in Nsp2 that was similar to those in BJEU06-1-like strains. Interestingly, GP3 in the SC202404 strain had a unique 25 aa premature termination at the C-terminus, and GP4 contained a consecutive deletion of 6 aa, which has been previously reported in TZJ226 isolated in 2020 in Henan Province [28]. Previous studies showed that the immune cell epitopes of PRRSV GP3 and GP4 play certain roles in immunity [11], however, since GP3 was demonstrated to be absent from the virions of PRRSV strain IAF-Klop, this protein may not be required for viral infection initiation [34]. Whether the premature termination in GP3 and the deletion in GP4 affect its ability to counter host immune reactions requires further study.

Although SC202404 was first identified in Sichuan Province in 2024, divergence-time estimations for TZJ226 and SC202404 strains showed that the SC202404 strain emerged approximately 4 years earlier than the TZJ226 strain. Moreover, a recombination analysis revealed that TZJ226 is a natural recombinant virus of TZJ2781 (major parental strain) and SC202404 (minor parental strain). Therefore, we speculated that the SC202404 strain likely spread from Sichuan to Henan Province and underwent recombination with the local strain TZJ2781, resulting in the recombinant strain TZJ226. However, due to the limited number of comprehensive genetic evolution data and regional sequences currently available, the exact geographical directionality and dynamics of this transmission cannot be definitively substantiated. Future large-scale phylogeographic and molecular epidemiological studies are required to accurately trace the transmission routes of the novel SC202404 strain. Our study aids in understanding the transmission and genetic evolution of the novel PRRSV-1 strain in China.

PRRSV-1 strains exhibit genetic diversity and cause highly variable clinical presentations [35]. Most Pan-European subtype 1 strains have low pathogenicity, resulting in mild clinical symptoms, in pigs. However, recently, highly pathogenic strains of subtype 1 have appeared in several countries, including Italy, Spain, and South Korea. In China, all the isolated PRRSV-1 strains belong to subtype 1 [14]. Currently, there is limited research on the pathogenicity of the Chinese PRRSV-1 strains, and only a few isolates have been evaluated for pathogenicity in pigs. The Chinese Amervac-like strains (GZ11-G1, HLJB1, and GD2022) and BJEU06-1-like strains (ZD-1, 181,187–2, SD1291, and ZJ01) have mild or moderate pathogenicity to piglets (Table 1). To test the pathogenicity of the novel PRRSV-1 isolate, five 4-week-old piglets were infected with the SC202404 strain. The SC202404-infected piglets developed a transient high fever (> 41.0 °C) and initial clinical signs such as lethargy and anorexia as early as 1 dpi. While such a rapid onset is faster than the typical incubation period associated with natural PRRSV-1 exposure, we attribute this to the intranasal inoculation method. The intranasal challenge route allowed a high titer of the virus (3 × 10^5.5^ TCID_50_/pig) to be delivered directly into the lungs, where it immediately infected the highly susceptible PAMs. This rapid, localized infection in the lungs likely triggered an acute inflammatory response and the subsequent early febrile reaction, which is observed in several animal experimental PRRSV-1 challenges [36–38]. Moreover, SC202404 could cause moderate hemorrhagic and interstitial pneumonia, and mild hemorrhages in the lymph nodes, spleen, and heart. Similarly, the lower pathogenicity of PRRSV-1 strain GD2022 could also cause obvious bleeding points in the heart and spleen of the challenged piglets [39]. All of the PRRSV-infected piglets survived. Although the SC202404 isolate caused a shorter fever period (2 days) compared to other BJEU06-like strains (3–6 days), it caused obvious clinical symptoms and pathological damage to multiple tissues in piglets, including lung, lymph nodes, heart, spleen, and kidney (Table 1) [37, 39–43]. Of note, the highest viral loads in the SC202404-challenged piglets were consistently detected in the heart across all five animals (Fig. 5c), which is consistent with the tissue tropism of PRRSV HuN2021 isolate [17]. Therefore, SC202404, a BJEU06-1-like strain, is a moderately pathogenic PRRSV-1 virus in piglets.

**Table 1:** Comparison of the pathogenicity of SC202404 strains and other strains in China

Strains	Isolation Date	Type	Country	The days of inoculation (dpi)	Pathogenicity in piglets	Accession No
SC202404	2024	BJEU06-1-like	China	14	Moderate hemorrhagic and interstitial pneumonia; lymph node, spleen, and heart hemorrhage, renal ecchymoses; reduced body weight; alveolar epithelial proliferation and moderate alveolar diaphragm widening in the lungs;worsening disease signs of diarrhea, muscle tremors, sneeze, anorexia, and mild ataxia 2 days fever (≥ 40 ℃) Moderately pathogenicity	PV533618
ZD-1	2016	BJEU06-1-like	China	21	Mild interstitial pneumonia; lymph node hemorrhage; alveolar epithelial proliferation and moderate alveolar diaphragm widening in the lungs; diffuse lymphocytic hyperplasia in the lymph nodes; high levels of viremia in the serum; and elevated viral loads in the lungs, lymph nodes, and tonsils; 6 days fever (≥ 40 ℃) Moderately pathogenicity	OP355712
181,187–2	2023	BJEU06-1-like	China	14	severe pulmonary consolidation and necrosis; lymph node hemorrhage; depression, anorexia and shivering; 3 days high fever (≥ 40.5 ℃) Moderately pathogenicity	OQ856755
ZJ01	2024	BJEU06-1-like	China	14	Mild interstitial pneumonia; lymph node hemorrhage; localized pulmonary hemorrhage; cause hyperviremia; 6 days fever (≥ 40℃) Moderately pathogenicity	/
SD1291	2022	BJEU06-1-like	China	21	Mild lung consolidation and interstitial pneumonia; coughing, anorexia, and diarrhea; 4 days fever (≥ 40℃) Mild pathogenicity	/
HLJB1	2015	Amervac-like	China	14	Mild multifocal lung lesions; lymph nodes and spleen enlarged; reddened conjunctiva, skin cyanosis, mild transient pyrexia, dyspnea,; 3 days fever (≥ 40℃) Mild pathogenicity	KT224385
GZ11-G1	2011	Amervac-like	China	21	Mild multifocal lung lesions 3 days fever (≥ 40℃) Mild pathogenicity	KF001144
GD2022	2022	Amervac-like	China	15	Obvious pneumonia, tissue consolidation and bleeding points on lung; exhibit respiratory symptoms such as sneezing and wheezing; Heart flaccid with myocardial bleeding points 1 day fever (≥ 40℃) Mild pathogenicity	OQ606399

The SC202404 isolate in this study presents an atypical and potentially attenuated phenotype compared to classical PRRSV-1 strains, such as the early fever, rapid viral clearance in serum, and high-level viral load in heart. Multiple unique amino acid mutations (GP3 and GP4) within the genome of SC202404 isolate might be an explanation for its atypical phenotypes in PRRSV-infected pigs. GP3 contains a hypervariable region located in the C-terminal end that overlaps with GP4 [44]. Previous studies indicated that this region is critical for mediating viral entry via interaction with the host receptor CD163 [45]. Importantly, GP4 is a major determinant of viral cellular tropism [46]. Therefore, these unique GP3/GP4 mutations may be potential mechanisms leading to virulence attenuation or phenotype transformation in the SC202404 isolate, which deserves further investigation using reverse genetics systems. Taken together, the PRRSV-1 SC202404 strain is characterized by a hyperacute early systemic phase, followed by rapid tissue distribution that drives subsequent humoral immunity in pigs, which merits special attention in control and vaccine strategies.

## Conclusions

In conclusion, a novel PRRSV-1 strain, SC202404, with unique molecular markers in GP3 and GP4, was isolated from Sichuan Province in 2024. Evolutionary and recombination analyses revealed that the SC202404 strain likely spread from Sichuan to Henan Province and underwent recombination with the local strain TZJ2781, resulting in the recombinant strain TZJ226. A further study indicated that SC202404 is a moderately pathogenic PRRSV-1 strain in piglets. Our study aids in understanding the genetic evolution of the PRRSV-1 strain and highlights the importance of preventing and controlling PRRSV-1 strains in China.

## Supplementary Information

Supplementary Material 1.

## Data Availability

The data used to support the findings of this study are included within the article. The PRRSV nucleotide sequence data of this study were submitted to GenBank database (http://www.ncbi.nlm.nih.gov/genbank/) under accession number: PV533618.
