# Supplementary material for: Genetic evolutionary and pathogenicity analyses of a novel porcine reproductive and respiratory syndrome virus 1 strain SC202404 that emerged in Southwestern China
Source: BMC Vet Res. 2026 Apr 25;22:335. doi: 10.1186/s12917-026-05488-7 (PMC13248377; doi:10.1186/s12917-026-05488-7)
Supplement: Supplementary file 1 — Supplementary Material 1. [file 12917_2026_5488_MOESM1_ESM.docx]

TABLE S1. Primers used for detection of SC202404 and the amplification of its full-length genome

| Fragment | Primer sequence (5’-3’) | Position in genome | Product size (bp) |
| --- | --- | --- | --- |
| 1 | ATGATGTGTAGGGTATTCCCCC  CACTTCTGCGGAACAACCTCCAA | 1-1962 | 1962 |
| 2 | CCTAGCGTCTGCTTACAGACTACC  AACGCCCCTGGGACACCACATA | 1847-4035 | 2189 |
| 3 | TGGTTCTATGCGGCGCGTTCG  ACCAGGGTGTGACCCATGCAA | 3716-6075 | 2360 |
| 4 | TTGGTTCTGGTCTTGTGACAAC  TGGATTATTTGCTTGGATAACTC | 5698-7764 | 2067 |
| 5 | GTGGAGGTAAAGAAATCAACTGA  AGCCACCTTCACCATGTTTAT | 7523-9727 | 2205 |
| 6 | GGAGGTACCAGTCCCGTCGAGG  GGCTGTTGCCGGTCCTATACAC | 9591-11410 | 1820 |
| 7 | AGAACTGCCCCACGCATTTAT  GTCAGTGTAGTCTTTGCCGTC | 11071-11621 | 551 |
| 8 | AGTTGGAAGGGCTCACGTGGTC  AGGCGAACGCCTCAGAAACC | 11304-12997 | 1694 |
| 9 | TATTATCACCACCAAATAGACGG  CTTCTCAGGCTTTTTCCTTTT | 12887-14740 | 2196 |
| 10 | TGCTAGGCCGCAAGTACAT  GGCCGTTGTTATTTGGCATA | 14,581-15,068 | 487 |

**Table S2**: Information of RT-qPCR primers targeting the NSP2 of PRRSV SC202404.

| Virus | Primer name ^a^ | Primers sequence (5´-3´) | Primer position | Length (bp) |
| --- | --- | --- | --- | --- |
| SC202404 | F | ACAGATGAAGCCACAGA | 2630-2646 | 272 |
| SC202404 | R | GACGCTACCCTCACTAA | 2885-2901 |  |
